# Comprehensive pathogen detection associated with four recurrent episodes of Kawasaki disease in a patient during a single year using next-generation sequencing

**DOI:** 10.1099/jmmcr.0.005019

**Published:** 2016-02-01

**Authors:** Hiromichi Hamada, Tsuyoshi Sekizuka, Kunihiro Oba, Harutaka Katano, Akiko Kinumaki, Masaru Terai, Tetsuya Mizutani, Makoto Kuroda

**Affiliations:** ^1^​ Department of Pediatrics, Tokyo Women's Medical University Yachiyo Medical Center, 477-96 Owada-Shinden, Yachiyo, Chiba 2768524, Japan; ^2^​ Laboratory of Bacterial Genomics, Pathogen Genomics Center, National Institute of Infectious Diseases, 1-23-1 Toyama, Shinjuku-ku, Tokyo 162-8640, Japan; ^3^​ Department of Pediatrics, Showa General Hospital, 2-450 Tenjin, Kodaira, Tokyo 187-8510, Japan; ^4^​ Department of Pathology, National Institute of Infectious Diseases, 1-23-1 Toyama, Shinjuku-ku, Tokyo 162-8640, Japan; ^5^​ Department of Pediatrics, Graduate School of Medicine, University of Tokyo, 7-3-1, Hongo, Bunkyo-ku, Tokyo 113-0033, Japan; ^6^​ Department of Virology 1, National Institute of Infectious Diseases, 4-7-1 Gakuen Musashimurayama, Tokyo 208-0011, Japan; ^7^​ Faculty of Agriculture, Tokyo University of Agriculture and Technology, 3-5-8 Saiwaicho, Fuchu City, Tokyo 183-8509, Japan

**Keywords:** cervical lymphadenopathy, conjunctival injection, injected lips/pharynx, IVIG+antibiotic treatment, Kawasaki disease, oedema/erythema of extremities, rash, vasculitis/fever

## Abstract

**Introduction:**

Kawasaki disease (KD) is the most common multisystem vasculitis in childhood. Pathogens can be associated with the onset of KD. However, a lack of consistency prevails among reports about this disease.

**Case presentation:**

For this case of a 1-year-old boy with four recurrent episodes of KD within a year, we analysed profiles of pathogen reads in his serum and pharynx specimens using next-generation sequencing. Comparative analysis of the identified bacterial reads from serum samples found significant correlation of bacteria such as *Streptococcus* and *Haemophilus* spp. with the first and fourth episodes (*R*^2^ = 0.9506) before treatment. In the first convalescent phase, the number of *Streptococcus* spp. was reduced remarkably (*P* < 0.0001). From sequencing of the pharynx specimen from the fourth episode, a similar correlation was found with serum from the fourth episode (*R*^2^ = 0.6633).

**Conclusion:**

In this case, *Streptococcus* spp. may have been associated with onset of KD. Further studies must be undertaken to evaluate the putative association of micro-organism infection with KD pathogenesis.

## Introduction

Kawasaki disease (KD) was identified in 1967 as an acute systemic vasculitis of infancy and early childhood (Kawasaki, 1967), but its aetiology remains unclear. Observations suggest infectious agents as candidates causing the onset of KD. In many KD cases, pre-existing infections have been found. Moreover, the resemblance of KD to toxic shock syndrome suggests a causative role of uncharacterized infectious agents. To date, several superantigen-producing bacteria including *Streptococcus pyogenes*, *Staphylococcus aureus* and *Yersinia pseudotuberculosis*, as well as viruses such as Epstein–Barr virus, have been implicated as agents causing KD (Leung *et al.*, 1993). Regarding the intestinal microbiome in KD, *Streptococcus* spp. were notably isolated from KD patients with an expanded T-cell population expressing Vβ2 (Nagata *et al.*, 2009). However, a lack of consistency prevails among reports.

These disparate findings suggest that the inflammation observed in KD is not the result of a single agent but rather is from various infectious agents in genetically susceptible individuals with an underlying immune system disorder. Results of genome-wide association studies suggest that some genetic susceptibility might be associated with the disease (Onouchi *et al.*, 2008).

Recently, next-generation sequencing (NGS) has enabled the determination of abundant sequences. This powerful technique, using an unbiased comprehensive approach, can identify sequences of possible pathogens in clinical specimens (Kuroda *et al.*, 2010, 2012; Takeuchi *et al.*, 2014).

We experienced the case of a boy with four instances of recurrent KD within a year. It is extremely rare to observe such recurrent KD. We analysed his serum and pharynx samples using direct sequencing with NGS, which is a novel and unbiased approach.

## Case report

**Fig. 1.**
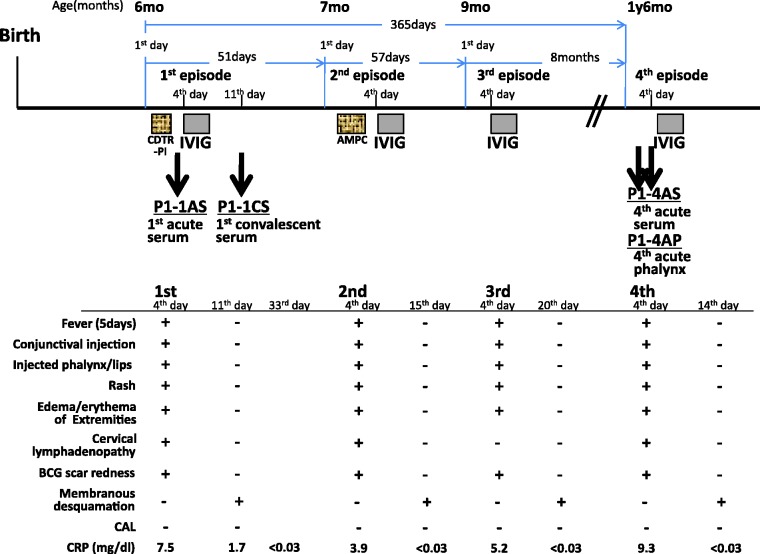
Schematic time course of the four episodes of recurrent KD in patient P1.+ and – respectively denote positive or negative for each clinical manifestation. Samples P1-1AS, P1-4AS and P1-4AP (see text) were collected on day 4 of the illness before IVIG treatment. P1-1CS was collected on day 11 of the illness: CAL, coronary artery lesion; CRP, C-reactive protein; IVIG, intravenous immunoglobulin.

We identified a KD patient (patient P1) who experienced four recurrent episodes of KD within a single year. Clinical investigations for all four events are presented in Fig. 1. In all four events, he fulfilled the diagnostic criteria of KD such as fever for 5 days, conjunctival injection, injected lips/pharynx, rash, oedema/erythema of his extremities and BCG scar redness (Fig. 1). Cervical lymphadenopathy was observed except in the third episode. In each convalescent phase, membranous desquamation was observed in the fingers. He presented cough and nasal discharge as well as fever before admission, except in the second episode. He took cefditoren pivoxil for 2 days before first admission, and amoxicillin for 3 days before the second admission (Fig. 1).

After each of the four events, he was admitted on the fourth day of illness. Laboratory data taken on each day of admission are presented in Table 1. Data at each admission were reasonable for KD. Intravenous immunoglobulin treatment (IVIG) was administered at a high dose, yielding a marked improvement of each of the four events within 48 h. We confirmed the end of each event by the fact that all symptoms improved and serum C-reactive protein levels fell below the detectable level ( < 0.03 mg dl^− 1^). The intervals between the respective episodes were 51 days, 57 days and 8 months (Fig. 1). He had no coronary artery lesion after any of these events.

**Table 1.** Blood test at each admission

Test	Day 4 of first episode	Day 4 of second episode	Day 4 of third episode	Day 4 of fourth episode
WBCs ( × 10^3^ μl^− 1^)	12.65	13.22	20.46	20.39
Neutrophils (%)	50	nd	68	84
Lymphocytes (%)	42	nd	27	12
PLTs ( × 10^4^ μl^− 1^)	30.2	25.9	27.0	33.6
TP (g dl^− 1^)	6.3	7.0	7.1	6.1
ALB (g dl^− 1^)	4	4.0	4.0	3.7
AST (IU l^− 1^)	31	59	162	32
ALT (IU l^− 1^)	23	33	156	54
Na (mEq l^− 1^)	136	134	133	132
CRP (mg dl^− 1^)	7.47	3.85	5.19	9.29
Group A *Streptococcus*antigen	nd	–	–	–
Adenovirus antigen	–	–	–	–
Blood culture	–	–	–	–

ALB, Albumin; ALT, alanine aminotransferase; AST, aspartate aminotransferase; CRP, C-reactive protein; nd, no data; Na, sodium; PLT, platelets; TP, total protein; WBC, white blood cells.

## Investigations

Samples P1-1AS (Patient 1 – episode 1acute-phase serum), P1-4AS (Patient 1 – episode 4acute-phase serum) and P1-4AP (Patient 1 – episode 4acute-phase pharynx) were collected on day 4 of illness before IVIG treatment (Fig. 1). P1-1CS (Patient 1 – episode 1convalescent-phase serum) was collected on day 11 when he was discharged. No samples were collected in the second and third episodes. No control samples were collected between the episodes.

The samples were stored at − 80 °C in a deep freezer until analysis. Total nucleic acids (DNA and RNA) were prepared from serum or a swab of the pharynx, with subsequent purification with a High Pure Viral Nucleic Acid kit (Roche). Briefly, the double-stranded cDNA was prepared from total RNA using a SuperScript Double-Stranded cDNA Synthesis kit (Invitrogen). A DNA sequence library was prepared from the mixture of original DNA and double-stranded cDNA using a Genomic DNA Sample Preparation kit (Illumina). All sequencing runs for 80-mer single-end reads were performed using an Illumina Genome Analyser IIx sequencing kit. The obtained short reads were analysed under a MePIC2 web server (PMID: 24451106), followed by taxonomic visualization using megan v.4.2.6 with the following parameters: minimum support, 1 hit; minimum score, 50 for the bacterial genus level. For statistical calculations, we used *Metastats*, a statistical method for comparing clinical metagenomic samples (White *et al.*, 2009).

**Fig. 2.**
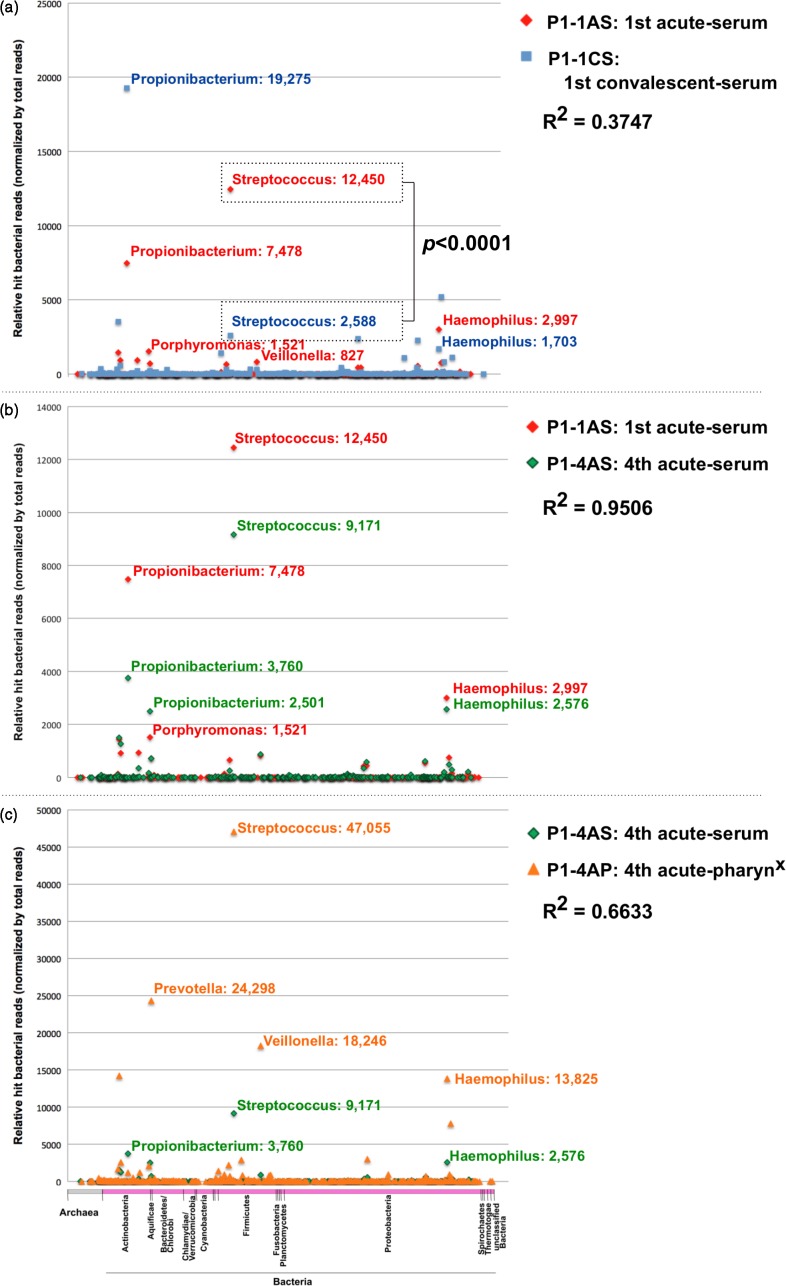
Dot plot of short-read numbers for archaea and all bacteria genera from the P1 specimens. Sequencing reads from the P1 specimens were analysed using a blastn homology search with a threshold score of 50.0. Similarity results were classified, respectively, into 11 and 653 genera of archaea and bacteria. Notable genera and the corresponding number of sequence reads are shown beside each dot for comparisons between P1-1AS and P1-1CS (a), P1-1AS and P1-4AS (b) and P1-4AS and P1-4AP (c).

Information from all obtained short reads and the blastn search results is presented in Table 2. Comparing serum specimens from the first acute attack (P1-1AS) and the first convalescent phase (P1-1CS), the most abundant bacterial sequences in the first acute-phase serum sample (P1-1AS) were *Streptococcus* spp., and these were reduced remarkably in the serum sample from the first convalescent phase (P1-1CS) (Fig. 2a). Between the P1-1AS and P1-1CS samples, a significant change was found in the number of bacteria between the acute and convalescent phases (*P* < 0.0001).

**Table 2.** Number of sequencing reads based on taxonomic classification Information is given for the sequencing reads for the specimens of patient P1. All analyses were run on 21 October 2009 and had a read length of 80 nucleotides.

Sample ID	Specimen	Kit version	Total sequencing reads	Human	Archaea	Bacteria	Virus	Not assigned	No hits
P1-1AS	First acute serum	SBS v3	7300184	98.8 %	0.00 %	0.36 %	0.00003 %	0.02 %	0.78 %
P1-1CS	First convalescent serum	SBS v3	4992086	97.6 %	0.00 %	0.52 %	0.00024 %	0.03 %	1.85 %
P1-4AS	Fourth acute serum	SBS v3	4890663	98.8 %	0.00 %	0.29 %	0.00022 %	0.03 %	0.90 %
P1-4AP	Fourth acute pharynx	SBS v3	6881957	97.0 %	0.00 %	1.66 %	0.00125 %	0.29 %	1.02 %

The bacterial genus classification of serum specimens from the first (P1-1AS) and fourth (P1-4AS) acute phases, which were collected before each IVIG treatment, showed that the most abundant bacterial sequences were from members of the genus *Streptococcus* in both specimens (Fig. 2b). The coefficient of determination for the total bacterial reads between the P1-1AS and P1-4AS samples was 0.9506, indicating that almost identical potential pathogens might be involved in the recurrent KD symptoms. Metagenomic NGS short reads do not support specificity sufficiently to clarify the detected bacteria species. Regarding viruses, 11 reads were similar to Torque teno virus and Torque teno-like mini virus, which is a ssDNA virus (circovirus).

Bacterial genus classification of the P1-4AS and P1-4AP samples, which were collected before IVIG treatment, showed that the most abundant bacterial sequences were from members of the genus *Streptococcus* in both specimens (Fig. 2c). The coefficient of determination from the total bacterial reads between the P1-4AS and P1-4AP samples was 0.6633. In particular, *Streptococcus* and *Haemophilus* spp. were significantly identified in both specimens, whereas anaerobic bacteria such as *Prevotella* and *Veillonella* were observed at a reduced level in serum (P1-4AS), indicating that serum-resistant bacteria such as *Streptococcus* spp. might become more prevalent than serum-sensitive bacteria. Regarding viruses in the fourth acute-phase pharyngeal swab sample (P1-4AP), one read was similar to Torque teno virus and Torque teno-like mini virus, whereas 85 reads (total 0.0012 %) were similar to human rhinovirus C.

## Discussion

Recurrent KD was identified in 3.5 % of all KD patients in a Japanese survey (Makino *et al.*, 2015). In the USA, the Centers for Disease Control and Prevention national KD surveillance (1984–2008) showed 1.7 % recurrent KD among the 5557 US KD patients (Maddox *et al.*, 2015). In such a population, a patient with recurrence four times within a year is extremely rare. Compared with non-recurrent KD patients, KD patients experiencing a recurrent KD episode are more likely to satisfy the atypical KD case definition. However, patient P1 had typical symptoms. We were able to diagnose the recurrence of KD easily and to confirm each onset day of the recurrence. To verify the end of each episode, we checked normal serum levels of C-reactive protein during the convalescent phase of each episode.

As described in this report, we demonstrated the detection of potential pathogens for patient P1 with KD using NGS. In this patient with four recurrent episodes of KD, comparative analysis of the identified bacterial reads suggested that *Streptococcus* spp. were involved in the acute phase of KD. In addition, samples from the convalescent phase showed different bacterial read profiles, whereas bacterial reads in the recurrent KD samples were notably correlated between the first and fourth attacks, implying that a similar causative agent(s) might be associated with recurrence. This study indicated that some bacterial sequences were identified in the patient's bloodstream, although conventional clinical testing indicated that all blood cultures were negative (Table 1). As described in the case presentation, antibiotics were prescribed for the first and second episodes before diagnosis of KD.

Previously, using a NGS and multivirus real-time PCR system, detection of possible pathogens from a cervical lymph node biopsy specimen from a boy with KD was reported. NGS identified *Streptococcus* spp. in the lymph node (Katano *et al.*, 2012). From metagenomic analysis, gut microbiota of KD patients also showed that the number of sequencing reads with similarity to *Streptococcus* spp. increased markedly during the acute phase in most patients (Kinumaki *et al.*, 2015). These results correlate with our results from patient P1. Streptococci include various pathogenic bacteria and probiotic bacteria that promote human health. Therefore, this additional species discrimination can elucidate aspects of the KD-associated microbiota.

A question arises about the source of these bacteria if the detection is reasonable. It is possible that bacterial foci exist other than in the blood. Fragmented bacterial nucleic acids were detected in the blood samples. The read sequences from the pharynx during the fourth episode showed a similar correlation to those from acute serum in the fourth episode, suggesting that commensal bacterial flora might leak into the bloodstream. In the acute phase of KD, significant production of TNF-α and vascular endothelial growth factor, or abnormal T-cell activation might contribute to the observed fragile mucosal membrane, which might cause leakage (Terai *et al.*, 2003). More evidence must be accumulated to assess these hypotheses.

Good correlation was found for *Streptococcus* and *Haemophilus* spp. but not for anaerobic bacteria such as *Prevotella* and *Veillonella* spp. *Streptococcus* and *Haemophilus* spp. possess serum resistance mechanisms to complement-mediated immunity using inhibitors of complement, complement peptidase, capsular polysaccharide and sialic acid (Jarva *et al.*, 2003). Such resistance characteristics contribute to bacteraemia, which might result in the increased detection of *Streptococcus* and *Haemophilus* spp. observed in this study.

This study had several limitations. First, this is a case report: only one patient has been described. We can only suggest that this methodology might have some power to investigate infection as an aetiology of KD. Secondly, no control samples were collected between the episodes for comparative purposes. Furthermore, no samples were collected during the second and third episodes. The probability exists that our findings were coincidental, for example relating to fluctuations in normal flora. Therefore, it is necessary to confirm that streptococcal DNA in particular is not a phenomenon associated with every infection/inflammation of the pharynx.

In conclusion, we have presented a case of a 1-year-old boy with four recurrent episodes of KD within a single year. From the use of unbiased NGS, this report presents profiles of the pathogen reads in his serum and pharynx specimens. In this case, *Streptococcus* spp. might be associated with onset of KD. However, further studies must be undertaken to confirm any association of micro-organism infection with the pathogenesis of KD.
